# Children's Country Hospitals

**Published:** 1888-06-09

**Authors:** A. R. Newman

**Affiliations:** Secretary. 10, Buckingham Street, Strand.


### CHILDREN'S COUNTRY HOSPITALS.

IN a recent article on the "Poor in Towns," you invite those engaged in the work of sending children into the country to give their experience, and I, as Secretary of the Children's Country Holiday Fund, avail myself of the opportunity of laying before your readers our scheme of work. The fund was started in 1884 as a bond of union between various isolated workers, and has grown so rapidly that nearly twelve thousand children were sent away during the summer of 1886. The plan of work is very simple: Children are selected by various local committees scattered all over London, and are despatched in parties to the care of responsible people in the country, who have found respectable cottagers willing to receive little guests between the ages of five and fourteen at the rate of 5*s*. a-week each. How greatly the holiday is appreciated is shown by the fact that parents' payments steadily increase, the average in 1886 being over 30 per cent., and the total amount received amounting to no less than £1,972 13*s*. 8½*d*., a sum in itself sufficient to send away 3,500 children. The general management of the fund is in the hands of a central council, consisting of representatives of local committees and six co-opted members. All subscriptions are paid into one common fund, money so obtained being granted by the central council according to the specific needs of local committees. By this plan poorer neighbourhoods are not overlooked in favour of wealthy parts, where the obtaining of funds is no difficulty; and in keeping one purse for the needs of the metropolis, the Country Holidays Fund is able to give every little Londoner an equal chance of the coveted holiday. I shall be most happy to give further information to anyone who may wish for it, and am always to be found at the address given below between eleven a.m. and three p.m. A. R. NEWMAN, Secretary.

10, Buckingham Street, Strand.

